# Histone modifications and traditional Chinese medicinals

**DOI:** 10.1186/1472-6882-13-115

**Published:** 2013-05-27

**Authors:** Hsin-Ying Hsieh, Pei-Hsun Chiu, Sun-Chong Wang

**Affiliations:** 1 Systems Biology and Bioinformatics Institute, National Central University, No. 300, Chungda Road, Chungli city 32001, Taiwan

**Keywords:** DNA methylation, Histone code, Chromatin structure, Traditional Chinese medicine, Yin-yang, Phylogenetics, Synergy, DNMT, HDAC, HAT, H3S10 phosphorylation, H3K4 methylation, H3K9 methylation

## Abstract

**Background:**

Chromatin, residing in the nuclei of eukaryotic cells, comprises DNA and histones to make up chromosomes. Chromatin condenses to compact the chromosomes and loosens to facilitate gene transcription and DNA replication/repair. Chemical modifications to the histones mediate changes in chromatin structure. Histone-modifying enzymes are potential drug targets. How herbs affect phenotypes through histone modifications is interesting.

**Methods:**

Two public traditional Chinese medicine (TCM) databases were accessed to retrieve the chemical constituents and TCM natures of 3,294 TCM medicinals. NCBI taxonomy database was accessed to build the phylogenetic tree of the TCM medicinals. Statistical test was used to test if TCM natures of the medicinals cluster in the phylogenetic tree. A public chemical-protein interaction database was accessed to identify TCM medicinals whose constituent chemicals interact with human histone-modifying enzymes. For each histone modification, a correlation coefficient was calculated between the medicinals’ TCM natures and modification modulabilities. Information of the ingredient medicinals of 200 classical TCM formulas was accessed from a public database.

**Results:**

It was found that 1,170 or 36% of the 3,294 TCM medicinals interact with human histone-modifying enzymes. Among the histone-modifying medicinals, 56% of them promote chromatin condensation. The cold-hot natures of TCM medicinals were found to be phylogenetically correlated. Furthermore, cold (hot) TCM medicinals were found to be associated with heterochromatinization (euchromatinization) through mainly H3K9 methylation and H3K4 demethylation. The associations were weak yet statistically significant. On the other hand, analysis of TCM formulas, the major form of TCM prescriptions in clinical practice, found that 99% of 200 government approved TCM formulas are histone-modifying. Furthermore, in formula formation, heterochromatic medicinals were found to team up with other heterochromatic medicinals to enhance the heterochromatinization of the formula. The synergy was mainly through concurrent DNMT and HDAC inhibition, co-inhibition of histone acetylation and H3S10 phosphorylation, or co-inhibition of H3K4 demethylation and H3K36 demethylation.

**Conclusions:**

TCM prescriptions’ modulation of the human epigenome helps elucidation of phyto-pharmacology and discovery of epigenetic drugs. Furthermore, as TCM medicinals’ properties are closely tied to patient TCM syndromes, results of this materia-medica-wide, bioinformatic analysis of TCM medicinals may have implications for molecular differentiation of TCM syndromes.

## Background

Histones are evolutionarily conserved proteins that abound in the cells of eukaryotes including plants and animals [1]. They form protein families and two copies of each of the structurally similar histones H2A, H2B, H3 and H4 assemble into histone octamers. DNA sequence wraps around the octamers to form nucleosomes, which constitute the subunits of chromatin. Nucleosome-nucleosome and histone-DNA interactions take place to tighten or loosen the chromatin structure, prohibiting or permitting access of the transcriptional machinery, such as RNA polymerase II and regulatory factors, to the DNA sequence. Gene activities and thus genomic functions can change independent of the DNA sequence. Chromatin structure is altered by covalent modifications to the amino acid residues in the unstructured tails of histones. For example, acetylation of the lysines in H3 and H4 N-termini neutralizes the otherwise positively charged histones, weakening the coupling between histones and negatively charged DNA sugar-phosphate backbone. The relaxing chromatin is associated with active gene transcription [2], so is cytosine hypomethylation, a covalent modification to the DNA that is found in association with histone acetylation [3]. An equally important property of histone modifications and DNA methylation is that modification patterns, once established, propagate through cell divisions. Different combinations of covalent modifications over the chromatin give rise to different cellular phenotypes. A histone code, supplementary to the DNA sequence, for cellular functions was therefore recently proposed [4].

Traditional Chinese medicine (TCM) has developed a system of theories and practices since at least 2,000 years ago and remains popular in some Far East Asian areas. In contrast to the reductionist approach of modern western medicine, TCM diagnoses a patient via inspection (e.g. tongue color), listening/smelling, questioning and palpation (e.g. pulse-reading) [5]. Emotional, mental and environmental factors are usually also taken into account. Outcomes of the diagnostics are summarized as TCM syndromes (called *Zheng* in TCM) which are usually classified under the eight outlines: yin or yang, internal or external, cold or hot, deficiency or excess [5]. Yin and yang in TCM refer, respectively, to the materialistic and functional qualities of the body (parts). External and internal indicate the origin or direction of syndrome development. Cold and hot are manifestations of the syndrome through metabolism and body heat. Deficiency means lack of activities, such as immunodeficiency, of the body organ(s). Two examples of TCM syndromes are Lung-Stomach-yin deficiency with excessive heat and concurrent yin-yang deficiency, both being commonly diagnosed by TCM in type II diabetic patients [6]. A major feat of TCM is that Chinese herbal formulas that counteract the TCM syndromes have been developed so that once the patient’s TCM syndrome is identified, the Chinese herbal formula specific to the syndrome is readily prescribed [7-9]. Due to its diagnostic system, TCM is considered a holistic, personalized yet less specific therapy compared to modern western medicine.

As histone modifications and cytosine methylation play a role in the activity of genes, aberration in the pattern of modifications to histones and DNA, called epigenome, can lead to disease. Indeed, increasing evidence for dysregulated epigenomes in developmental, autoimmunological, metabolic and neurodegenerative disorders has been reported [10-13]. In particular, region-specific hypermethylation over a hypomethylated genome is characteristic of cancer cells [14]. Drugs that target the altered epigenome for cancer treatment have been under investigation. For example two compounds (vorinostat and romidepsin) that inhibit histone deacetylases have been FDA-approved for cutaneous T-cell lymphoma. As histone modifications and DNA methylation are evolutionarily conserved regulatory mechanisms for cell functions, chemicals, e.g. secondary metabolites, in plants and animals that modulate the human epigenome may be found. It is therefore not surprising that a recent study, of bioinformatic nature yet at the pharmacopeia scale, found 30% of ~3,000 traditional Chinese medicinals, the majority of which are herbs, were human epigenome-interacting [15]. Human epigenome-interacting herbs were extensively utilized in TCM formulas so that 99% of the studied formulas were epigenome-interacting. Furthermore, the epigenome-interacting herbs were found to serve in the formulas as the major herbs, called *Monarch* in TCM herbalism, that target patient’s main syndrome [15].

While modern western medicine remains the first choice for most patients, TCM, and other traditional medicines, has long been regarded as preventive or alternative for individuals at sub-healthy conditions or as complementary for patients at terminal conditions. As personalized medicine starts gaining impetus in modern western medicine [16], TCM has long been practicing tailored treatment, which is exemplified by the many possible different TCM syndromes that can be diagnosed to patients of a same disease. A medicine that is integrative of modern western medicine’s pathway-targeting and TCM’s epigenome-modulating therapy will be beneficial to patient’s well-being. A better understanding of TCM syndromes is essential. The diagnosed TCM syndrome of an individual can remain stable beyond the time span of a cell cycle. Evidence from recent studies demonstrates that histone modifications faithfully predict cell fates in various cellular contexts [17]. A link between TCM syndrome and epigenomic change can therefore be hypothesized. In an attempt to address the hypothesis, we propose to look at TCM medicinals first because of the following two observations: 1) TCM doctrine dictates that TCM syndromes (e.g. hot) should be treated by the counteracting (cold) medicinals [7]; and 2) the cold-hot annotations of most TCM medicinals are available as they have been characterized and documented ever since the first TCM materia medica 2,000 years ago [18]. We undertook the analysis in the following way. We first tested if the cold-hot and yin-yang TCM properties are, in a modern biological sense, viable through their relatedness in the phylogenetic tree of the medicinals. We next identified, using public chemical-protein interaction resources, the TCM medicinals whose chemical ingredients modulate DNA methylation and histone modifications of human cells. The TCM medicinals’ modulabilities of histone marks, which open, condense, or poise chromatin, were then correlated with their TCM properties. A significant correlation would lend support for the proposition of a link between epigenome patterns and TCM syndromes. We went on to study any synergistic effect of combing TCM medicinals in forming TCM formulas, under the perspective of chromatinization. Our findings may have implications for herbal pharmacology and epigenetic therapy.

## Methods

### Public databases and resources

Information about Chinese medicinals, including their TCM properties, constituent chemicals and scientific names in binomial nomenclature, was obtained from two open-access TCM databases. The Shanghai TCM database (http://www.sirc-tcm.sh.cn/en/service_1_1_1.html) at Shanghai Tcm Data Centre is funded by Shanghai municipal government and contains information of 8,896 traditional Chinese medicinals. We further supplemented the TCM information from a Singaporean TCM database (http://tcm.cz3.nus.edu.sg/group/tcm-id/tcmid.asp) containing 1,313 TCM medicinals [19]. The NCBI taxonomy database (http://www.ncbi.nlm.nih.gov/Taxonomy/taxonomyhome.html/) incorporates the phylogenetic knowledge from a variety of sources and contains 201,995 species from plants, animals and fungi [20]. A phylogenetic tree of Chinese medicinals was then built, consisting of 1,208 Chinese medicinals, where 95% of them are plants. The chemical-protein interaction database STITCH 2 (http://stitch.embl.de/) [21] contains 897,803 pairs of chemical-proteins associations between 14,732 human proteins and 53,092 chemicals. Information of 200 government-approved TCM formulas is available at http://www.ccmp.gov.tw/en/information/formula.asp. We converted the CAS registry numbers, for chemical identification, used in the TCM databases to the PubChem IDs used in the STITCH 2 database by the use of the NCBI PubChem server (http://pubchem.ncbi.nlm.nih.gov/).

### Data analysis

#### Phylogenetic correlation of TCM properties

The TCM nature of a medicinal can be cold (−3), mild cold (−2), cool (−1), neutral (0), mild warm (1), warm (2) and hot (3) according to the annotations in the TCM database. We assigned a score from −3 to 3 corresponding to the seven TCM natures from cold to hot as shown by the numbers in the parentheses. The TCM flavour of a medicinal can be sweet (+1), mild sweet (+0.5), pungent (+1), mild pungent (+0.5), plain (+1), sour (−1), mild sour (−0.5), bitter (−1), mild bitter (−0.5), salty (−1) and mild salty (−0.5) and their combinations. For example, if an herb is both sweet and mild pungent, its TCM flavour score is 1 + 0.5 = 1.5. The yin-yang score of a medicinal is then the sum of its TCM nature score and the TCM flavour score. The resulting cold-hot scores, ranging from −3 to 3, and yin-yang scores, ranging from −4 and 4, are symmetrically distributed. Additional file 1: Figures S1-S3. (Note that when a medicinal appears more than once in the TCM database, which is possible, although not frequent, due to the medicinal’s different effects from its different parts, we use the TCM annotation that first appears in the TCM database.) Evolutionarily relatedness of the cold-hot or yin-yang of the TCM medicinals is examined by the Moran’s *I* coefficient [22],

(1) $$I=\frac{N}{\sum_{i,j=1}^{N}w_{\mathrm{ij}}}\frac{\sum_{i,j=1}^{N}w_{\mathrm{ij}}\left(y_{i}-Y\right)\left(y_{j}-Y\right)}{\sum_{i=1}^{N}{\left(y_{i}-Y\right)}^{2}},$$

where *y*_*i*_ is the cold-hot or yin-yang score of medicinal *i*, *Y* the mean score of all the *N* medicinals, *w*_*ij*_ a weight that is inversely proportional to the distance *d*_*ij*_ between medicinals *i* and *j* on the phylogenetic tree: *w*_*ij*_ = 1/*d*_*ij*_. A *P*-value of the observed *I* can be calculated from the null distribution of *I*’s assuming no phylogenetic correlation of *y*’s. Moran’s *I* and *P*-value were calculated by the Moran.I function and *d*_*ij*_ by the cophenetic function in the R package ape [23].

#### Determination of histone-modifying TCM medicinals

The human enzymes that are responsible for the histone modifications under study are listed in the Additional file 1: Table S1. The residue-specific modification annotations were through links from Ensembl Human to Entrez Gene databases. A medicinal that modifies histone modification *X* if it contains chemicals that interact with the human enzymes responsible for the *X* modification, where the chemical-enzyme interaction was from the STITCH 2 chemical-protein interaction database [21]. Note that an interaction in STITCH 2 can be either activation, inhibition or not specified. In the case of not specified, we assumed it to be an inhibition, which is usually the case for chemical protein interactions. The *X* potency of the medicinal is the total number of chemical-protein interaction pairs that modify the *X* modification.

#### Unsupervised hierarchical clustering of the TCM medicinals

A medicinal is represented by an array of 18 attributes, that is, the potencies to modify the 18 histone marks. Comparison between the medicinals is then made by the Euclidean distance between the attribute arrays. Hierarchical clustering works by firstly placing the two most similar medicinals together, followed by merging the two into a ‘medicinal’. The pairing and merging are repeated on the remaining *N*–1 medicinals till all the medicinals are merged. Clustering of the TCM formulas was conducted the same way with the attribute array of a formula from the sum of the attribute arrays of the medicinals composing the formula. *N* is 1,170 or 199, the number of histone-modifying medicinals or formulas. We performed the hierarchical clustering by the heatmap function in R with default parameter settings.

## Results

### Positive phylogenetic correlation of the cold-hot/yin-yang TCM properties

**Figure 1:**
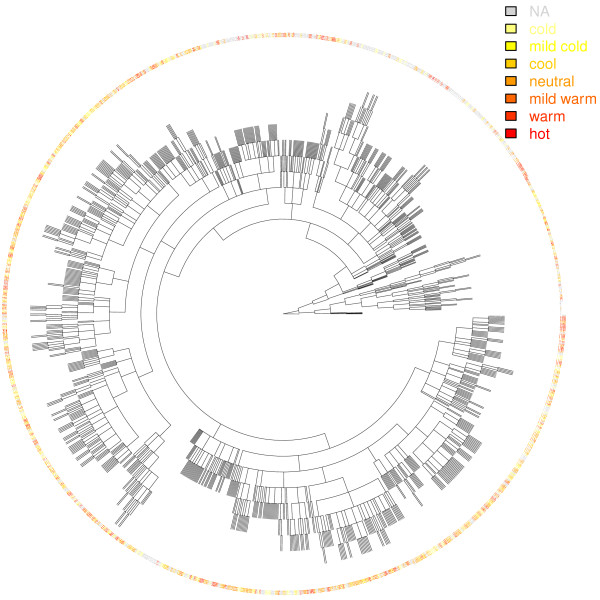
**Phylogenetic tree of 1,208 Traditional Chinese Medicinals.** Among the 3,294 TCM medicinals, 1,208 have entries in the NCBI taxonomy database. The scientific names of the medicinals, encompassing fungi, birds, snakes, …, and citrus, are replaced by numbers from 1 to 1208 counterclockwise from the right middle. Colors of the labels are linearly proportional to the cold-hot TCM natures of the medicinals so that the lighter the color the colder the medicinal. NA means the medicinal does not have a cold-hot annotation in the TCM database. Clustering of colors along the circle indicates positive phylogenetic correlation of the medicinals’ cold-hot property. Additional file 1: Figure S4 is the same figure with scientific names and in a vector format so that the image can be magnified indefinitely to read the names.

Among the basic TCM syndromes, coldness or hotness most easily discern themselves. For example, a healthy individual may be able to determine the coldness or hotness of her constitute by answering a standardized questionnaire. Yin-yang is less straightforward. However TCM asserts that sour, bitter and salty medicinals, believed to be, respectively, physiologically astringing, purging and moistening, are yin medicinals and that sweet medicinals and pungent medicinals, being nourishing and dispersing respectively, are yang medicinals [7,18]. If one prefers sweet foods, then her constitute is likely more yin (or yang-deficient). Furthermore, cold medicinals are yin and hot medicinals are yang. In Methods, we quantified the cold-hot annotation of each TCM medicinal by assigning a seven-level cold-hot score to it. We colour-code the medicinals based on their cold-hot scores and plot, in Figure 1, the distribution of the colours on the phylogenetic tree of the medicinals. Local clustering of the colours indicates that similar TCM properties are shared among evolutionarily related medicinals. Since evolutionary relatedness indicates similarity in the organisms’ biology including their metabolomes, local clustering of the TCM properties attributable to the metabolites in the medicinals is what one would expect. We employed Moran’s *I*[22], a statistical test for autocorrelation of adjacent phenomenon, and found a significant local clustering of the TCM cold-hotness on the phylogenetic tree (*P* = 6.4 × 10^-7^).

Likewise, after quantifying the yin-yang of the TCM medicinals, we colour-coded the tree in Additional file 1: Figure S5 and Additional file 1: Figure S6. Eyeball inspection and statistical test of the tree revealed a significant local clustering of the yin-yang on the tree (*P* = 1.3 × 10^-6^). In sum, the phylogenetic analysis of the two basic TCM properties supports TCM’s classification of medicinal properties, which would in turn support TCM’s classification of pathological states, through the TCM doctrine of syndrome-prescription mapping. After substantiating TCM medicinals’ effects, specifically the cold-hot and yin-yang, we are in a position to elucidate the underlying molecular mechanisms.

### Thirty-six percent of TCM medicinals are histone-modifying with over half of them chromatin-condensing

In TCM theory, perfect health is in yin-yang balance. However, the innate constitute of an individual can be slightly yin (e.g. cold) or yang (hot). Once the balance is broken (by inherited predisposition or environmental stress), a TCM syndrome develops. As the affliction progresses, the TCM syndrome can change its grade or to another TCM syndrome until it subsides under proper TCM treatment. The phenomenon of slow yet long-term effects of Chinese herbs and reversible dynamics of TCM syndrome development incites a link between Chinese herbalism and epigenetics [15].

**Figure 2:**
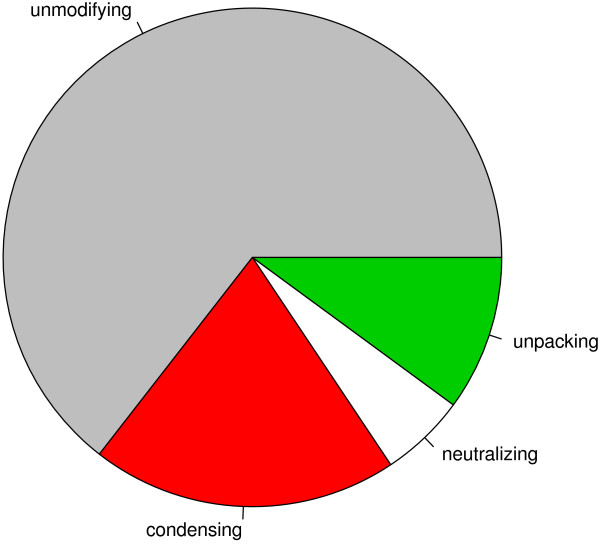
**Proportion of histone-modifying TCM medicinals among the 3,294 TCM medicinals.** Histone-modifying medicinals are considered chromatin unpacking if they cause more euchromatic modifications than heterochromatic modifications, etc.

In Table 1, we compile a list of histone modifications, including the ‘writers’ and ‘erasers’ of the modifications and the chromatin conformations resulting from the modifications. Histone methylation can give opposing chromatin structure depending on the site of methylation. Phosphorylation at serine 10 of H3 was found associated, in interphase, with rapid transcriptional activation in response to extracellular cues via the MAP kinase cascade. The mechanism of activation is not clear but is thought to be related to the histone acetylation at nearby lysines [28]. Also included in Table 1 are ATP-dependent chromatin remodelling complexes which alter chromatin structure via changes in the mobility of nucleosomes along the DNA. To study if a TCM medicinal changes the modification, we check if any chemical in the medicinal interacts with the human enzymes that catalyse (or inhibit) the modification. The more the chemicals that interact with the more the enzymes in the protein family for the modification, the more potent the medicinal is to change the modification. The result in Figure 2 indicates that 1,170 or 36% of the 3,294 medicinals are able to modify histone marks.

**Table 1:** Histone modifications and the associated chromatin conformation

**Histone/DNA modification**	**Enzymes**	Position/abbreviation^*^	**Conformation**	**Reference**
cytosine methylation	DNA methyltransferase	DNMT	heterochromatic	[24]
histone acetylation	histone acetyltransferase	HAT	euchromatic	[2]
histone deacetylation	histone deacetylase	HDAC	heterochromatic	[2]
histone methylation	histone methyltransferase	H3K4	euchromatic	[25]
		H3K36	euchromatic	
		H3K79	euchromatic	
		H3R17	euchromatic	
		H3K9	heterochromatic	
		H3K27	heterochromatic	
		H4K20	heterochromatic	
histone demethylation	histone demethylase	H3K4i	heterochromatic	[26]
		H3K36i	heterochromatic	
		H3R2i	heterochromatic	
		H4R3i	heterochromatic	
		H3K27i	euchromatic	
histone phosphorylation	mitogen- and stress-activated protein kinase (MSK), I kappa B alpha kinase (IKK)	H3S10	euchromatic	[27]
histone de-phosphorylation	MAP kinase phosphatase (MKP)	H3S10i	heterochromatic	[28]
ATP-dependent chromatin remodeling	ATP-dependent chromatin remodeling complex	ATP	hetero- or euchromatic	[29]

^*^K Lysine, *R* Arginine, S serine, *H3K4* H3K4 methylation, *H3K4i* H3K4 demethylation, *H3S10* H3S10 phosphorylation, *H3S10i* H3S10 dephosphorylation.

**Figure 3:**
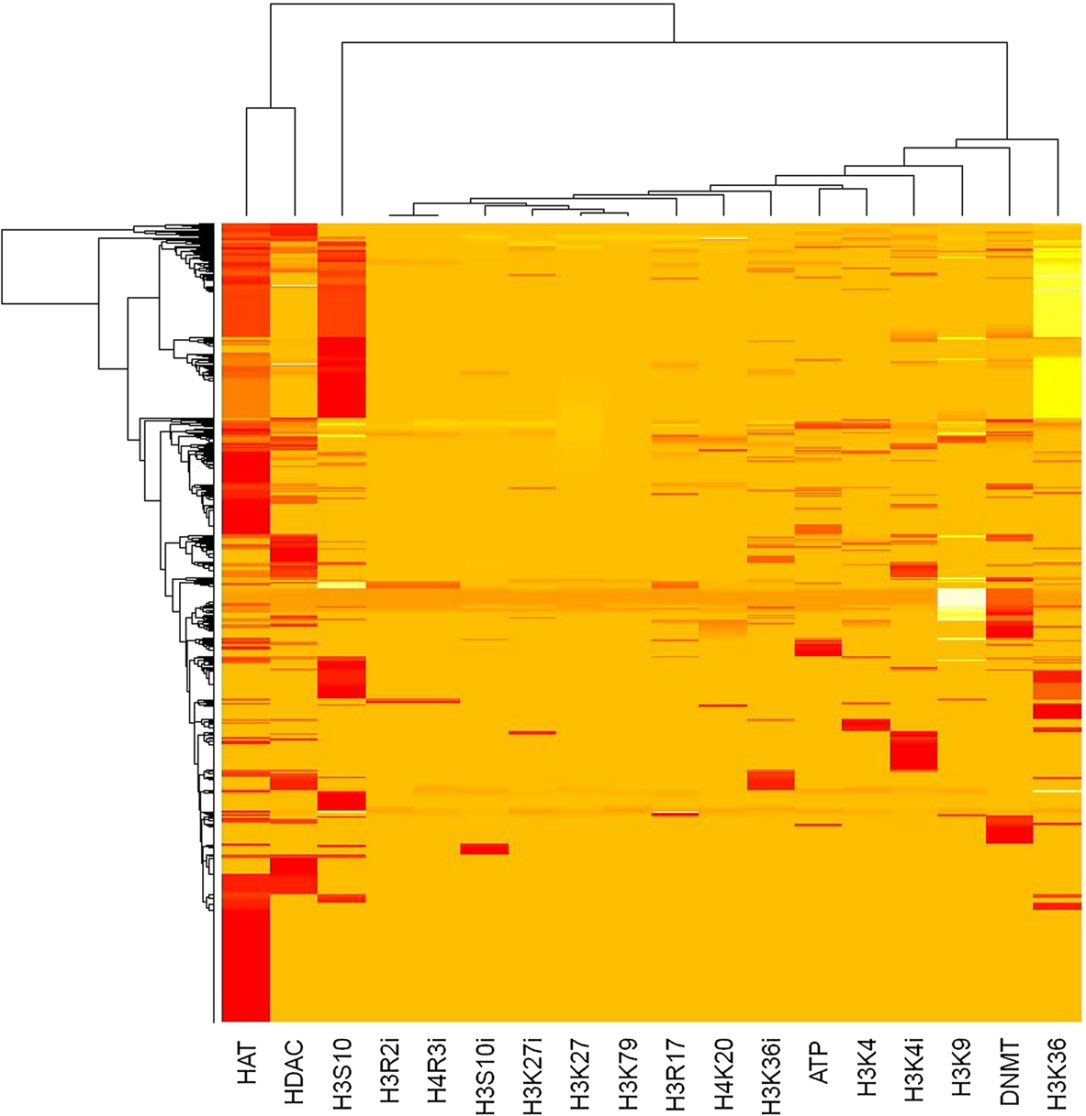
**Hierarchical clustering of the 1,170 histone-modifying TCM medicinals and the 18 modifications.** A row represents a medicinal and a column a modification. The lighter (darker) the shade, the more potent the medicinal is to activate (inhibit) the modification. Modifications are close within clades in the upper dendrogram if they are similarly utilized among the medicinals. Refer to Table 1 for the abbreviations of the modifications.

Histone code is redundant in the sense that different modifications on the same histone tail can result in the same chromatin conformation (rf. Table 1). To study the modes (or groups) of histone modifications that are employed by the TCM medicinals, we place the modifications closer in Figure 3 if they are more similarly utilized by the medicinals (see the hierarchical clustering in Methods). The resulting dendrogram on the top of Figure 3 unravels three distinct groups of modifications with histone acetylation separating from the rest, which further divides into histone phosphorylation and histone/DNA methylation. An interpretation of the divisions is that they reflect involvement of different coreactants, e.g. acetyl-CoA, ATP or SAM, in the disparate biochemical pathways of covalent histone modifications. Furthermore, in Figure 3, the non-covalent, ATP-dependent chromatin remodelling mechanism is found clustered with H3K4 methylation, a result that is consistent with the experimental finding of a direct coupling between H3K4 trimethylation and ATP-dependent chromatin remodelling in maintaining gene expression during development [30]. Altogether, the results endow us with confidence in the databases and research approach.

Histone modifications exert their functional importance through chromatin conformation. If a medicinal brings about more chromatin unpacking than condensing modifications, the medicinal is called chromatin unpacking. If a medicinal effectuates an equal number of unpacking and condensing marks, it is chromatin neutral (or poising). Figure 2 shows that among the 1,170 histone-modifying medicinals, 56%, 28% and 16%, respectively, condense, unpack and poise chromatin.

### Cold- and yin-TCM medicinals are associated with chromatin condensing while hot- and yang-TCM medicinals with chromatin unpacking

For each of the 18 modifications in Table 1, we correlate the modifying potencies of the 1,170 TCM medicinals with their cold-hot scores which were defined previously for the phylogenetic analysis. At the significance threshold of 0.05, we found cold-hot score to be negatively correlated with H3K9 methylation (Pearson correlation coefficient *r* = −0.07, *P* = 0.038) and H3K4 demethylation (*r* = −0.06, *P* = 0.049), both of which are modifications that condense chromatin. Since the higher the score, the warmer the medicinal (as defined in Methods), the result indicates that cold medicinals condense chromatin.

Similarly, for each of the histone modifications under study, we correlate the modifying potencies of the 1,170 TCM medicinals with their yin-yang scores defined previously for the phylogenetic tree of Additional file 1: Figures S5 and Additional file 1: Figure S6. At the significance threshold of 0.05, we found yin-yang score to be negatively correlated with H3K4 demethylation (*r* = −0.08, *P* = 0.012), which is a chromatin condensing modification. In other words, yin TCM medicinals condense chromatin.

When we redefined the yin-yang scores by using the TCM flavours only (i.e. without considering the cold-hot natures) and repeated the correlation analysis, we found, at the significance threshold of 0.05, yin-yang scores to be negatively correlated with H3K36 methylation (*r* = −0.07, *P* = 0.020) and H3K4 demethylation (*r* = −0.07, *P* = 0.033) and positively correlated with H3S10 phosphorylation (*r* = 0.07, *P* = 0.029). The result is again in concordance with the picture of a chromatin condensing effect of yin TCM medicinals.

### Similar medicinals combine in a TCM formula to reinforce the hetero- or euchromatinization

A TCM syndrome, once diagnosed, is readily prescribed a TCM formula consisting of multiple medicinals. The syndrome-formula mapping has been developed and documented by TCM healers through empiricism for thousands of years [7-9]. To study how formulas evolved, we analysed 200 government-approved and insurance-covered TCM formulas whose composing medicinals are known. The analysis was straightforward in that we worked on individual formula, instead of medicinal, as an entity. Additional file 1: Figure S7 shows the distribution of histone-modifying medicinals among the medicinals which make up the 200 formulas. Additional file 1: Figure S8 shows the clustering of the histone modifications employed by the medicinals composing the 200 formulas. The figures indicate that although the proportion of histone-modifying medicinals (Additional file 1: Figure S7) is higher in the set of medicinals making up the formulas, the modes of histone modifications by them (Additional file 1: Figure S8) are no different from those by the universe of 1,170 medicinals (Figure 3). The 200 TCM formulas can thus be considered representative in the sense that the medicinals in them are not epigenetically peculiar.

**Figure 4:**
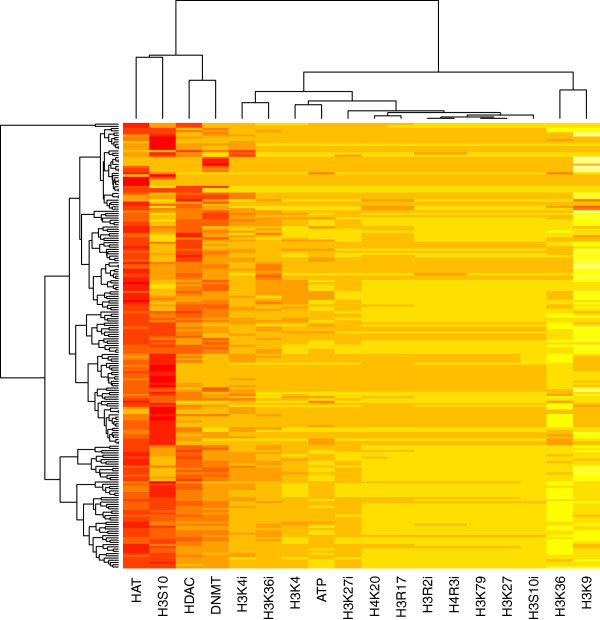
**Hierarchical clustering of the 199 histone-modifying TCM formulas and the 18 modifications.** A row represents a formula and a column a modification. The lighter (darker) the shade, the more potent the formula is to activate (inhibit) the modification. Modifications are close within clades in the upper dendrogram if they are similarly utilized by the formulas. Refer to Table 1 for the abbreviations of the modifications.

If a TCM formula is histone-modifying given that at least one of its composing medicinals is histone-modifying, we found that 199 out of the 200 formulas are histone-modifying. The dendrogram on the top of Figure 4 shows how histone modifications by the 199 histone-modifying formulas cluster. The result of the current *in silico* study indicates that many TCM formulas function as inhibitors of histone (de)acetylation, histone phosphorylation and DNA methylation. Furthermore, Figure 4 shows that formulas modulate the epigenome by: 1) co-inhibition of HAT and H3S10-phosphorylation; 2) co-inhibition of DNMT and HDAC; and/or 3) co-inhibition of H3K4-demethylation and H3K36-demethylation. Since DNMT and HDAC are both associated with heterochromatin, while HAT and H3S10-phosphorylation are both associated with euchromatin, so are H3K4-demethylation and H3K36-demethylation, the finding suggests a synergy among the medicinals in a formula to enhance the designated chromatin state.

**Figure 5:**
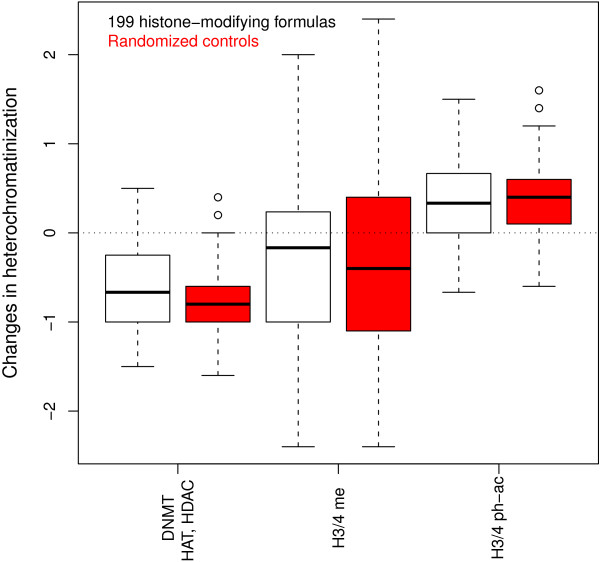
**Difference in the heterochromatinization between a formula and its composing medicinals.** A medicinal’s heterochromatinization is from the net number of heterochromatic modifications (me for methylation, ph for phosphorylation and ac for acetylation) it makes. A formula’s heterochromatinization is from the net number of heterochromatic modifications that the chemicals in the composing medicinals make. The difference should be compared to that from the 200 simulated control formulas (in red) that are similar to the real formulas in terms of median number of composing medicinals and median number of composing medicinals that are histone-modifying, except that the medicinals are randomly selected from the pool of medicinals making up the 199 real formulas. The closer the while boxes to zero than the red boxes indicates that, when formulas are formed, changes in the heterochromatic property are less than expected.

In another way of studying synergy, we compared the heterochromatinization of the individual medicinals in a formula with that of the formula as a whole. The result in Figure 5 shows lesser changes in the heterochromatinization before and after formulas are formed in comparison to 199 control formulas that are formed by randomly selecting *N* medicinals from the pool of medicinals making up the 199 formulas (*N* is the median number of medicinals calculated from the real formulas). The less mixing of counteracting chromatinization medicinals in the formulas than in controls suggests that heterochromatic formulas are formed by heterochromatic medicinals instead of by a very potent heterochromatic medicinal plus minor euchromatic medicinals. Taken together, the synergy analysis suggests that TCM formulas are formed by similar medicinals to reinforce the histone modifications of the component medicinals.

## Discussion

TCM natures (i.e. cold, cool, warm and hot) and flavors (i.e. pungent, sweet, sour, bitter and salty) have been essential properties of Chinese herbs since the early days of TCM in that the properties are closely connected to the classes of TCM syndromes they are supposed to treat. For example, yin-deficient physical constitutes and TCM syndromes are treated with yin-nourishing herbs. Similarly, cold-inclined individuals will be prescribed with warm herbs where cold can be considered warm-deficient. It is therefore of paramount importance that the TCM properties live up to the expectations of modern biology. Evolutionarily closely related species, such as Asian ginseng (*Panax ginseng*) and American ginseng (*Panax quinquefolius*), have similar genomes and may be expected to deliver similar pharmacological effects to the human body compared with herbs of dissimilar genomes. Since the nucleic acid or amino acid sequences are of a modern discovery, results of the phylogenetic analysis argue that TCM cold-hot and yin-yang are qualified properties for scientific investigation. Indeed, an *in vitro* study reported bitter, sour (yin) herbs to be associated with higher anti-oxidant activity than pungent, sweet (yang) herbs [31].

In the current computational study, we showed that more than one third of TCM medicinals and almost all TCM formulas are potentially histone-modifying. We went on to elucidate the association between the chromatinization of the histone modifications and the yin-yang of the TCM medicinals. TCM cold-hot and yin-yang are among the fundamental classifications of TCM syndromes on patients. Genome-wide profiling of histone modifications can be achieved nowadays by such high throughput techniques as chromatin immunoprecipitation followed by high-density chip hybridization (ChIP-chip) or high-throughput sequencing (ChIP-seq) [32,33]. An association between chromatin conformations and TCM syndromes provides a quantitative measure for TCM syndrome diagnosis which has traditionally been more qualitative and thus subjective [34,35]. Normalization and standardization of the TCM syndromes within a disease have been a top priority within the TCM community since the 1980s [36]. A metabolomic marker identification of a TCM syndrome in rats was recently reported [37]. Although both epigenome and metabolome can represent cellular conditions, transcriptome is more often considered cause of the phenotype. Indeed, a network-based study identified hormones and immune factors as predominant players in cold and hot TCM syndromes [38]. It was demonstrated that, in various cell lines, gene expression could be predicted by chromatin features [17,39]. Thus, our suggestion of a connection between chromatin conformations and TCM syndromes has impacts on TCM syndrome research. Further studies in this avenue are urged.

Herbs and formulas differ from conventional drugs in their multi-compound composition. The effects of many of the compounds on cells are, however, unclear, let alone studies of their interactions. By focusing on histone modifications and chromatin conformations the herbs impart, we showed that herbs in a formula cooperate to condense or unpack the chromatin and that a chromatin condensing/unpacking formula is composed of herbs that are chromatin condensing/unpacking. The finding is in agreement with a recent study in which warm (cold) herbs were found to connect with other warm (cold) herbs in the herb network constructed from TCM formulas [40]. The findings may shed light on herb combination rules for the development of complementary and new herbal prescriptions for such complex and emerging disorders as cancer and SARS.

Our study relied on open-access databases, namely the NCBI taxonomy database, Shanghai and Singapore TCM databases and German chemical-protein interaction database. Simple yet naive assumption about the medicinal chemicals’ reaching the nuclei was also made. Albeit the limitations, the materia-medica-wide bioinformatic approach taken here helps yield insights that can prove difficult to gain otherwise.

## Conclusions

With the data of 3,294 TCM medicinals from public resources and with the help of a chemical-protein interaction database, we found that 36% of the medicinals interact with human histone-modifying enzymes. Among the histone-modifying medicinals, 56% of them condense chromatin. Further exploration of the connection between histone modifications and TCM medicinals demonstrated that the cold-hot nature of TCM medicinals, one of the central properties of TCM, is phylogenetically correlated. Cold or yin (hot or yang) medicinals were then found to be associated with heterochromatinization (euchromatinization) through mainly H3K9 methylation and H3K4 demethylation. Studies of TCM formulas found that 99% of 200 government approved TCM formulas are histone-modifying. Furthermore, in formula formation, medicinals are combined in a way that heterochromatic medicinals team up with other heterochromatic medicinals to enhance the heterochromatinization of the formula. TCM prescriptions’ modulation of the human epigenome helps elucidation of TCM pharmacology and discovery of epigenetic drugs.

## Abbreviations

TCM: Traditional Chinese medicine; K: Lysine; R: Arginine; S: Serine; H3K4: H3K4 methylation; H3K4i: H3K4 demethylation; H3S10: H3S10 phosphorylation; H3S10i: H3S10 dephosphorylation.

## Competing interests

The authors declare that they have no competing interest.

## Authors’ contributions

HYH retrieved the data and performed the analysis. PHC participated in the analysis. SCW designed the study, analyzed the data and drafted the manuscript. All authors read and approved the final manuscript.

## Pre-publication history

The pre-publication history for this paper can be accessed here:

http://www.biomedcentral.com/1472-6882/13/115/prepub

## Supplementary Material

Additional file 1: Table S1 Enzymes that catalyze the modifications in human cells. **Figure S1**: The distribution of TCM medicinals’ yin-yang scores from their TCM natures. **Figure S2**: The distribution of TCM medicinals’ yin-yang scores from their TCM flavors. **Figure S3**: The distribution of TCM medicinals’ yin-yang scores from their TCM natures and flavors. **Figure S4**: Phylogenetic tree of the 1,208 TCM medicinals. The same plot as Figure 1 in the text except that the labels show the medicinals’ scientific names. **Figure S5**: Phylogenetic tree of the 1,208 TCM medicinals. The same plot as Figure 1 in the text except that the colors code for yin-yang of the TCM medicinals. The darker the shade, the more yang the medicinal. **Figure S6**: Phylogenetic tree of the 1,208 TCM medicinals. The same plot as Additional file 1: Figure S2 except that the labels show the scientific names of the TCM medicinals. **Figure S7**: Proportion of the histone-modifying medicinals among the 230 medicinals that make up the 200 TCM formulas. **Figure S8**: Hierarchial clustering of the 116 histone-modifying TCM medicinals in the 200 TCM formulas and the 18 histone modifications. Refer to **Table S1** for the abbreviations of the modifications. Click here for file
