# Supplementary material for: Histone modifications and traditional Chinese medicinals
Source: BMC Complement Altern Med. 2013 May 27;13:115. doi: 10.1186/1472-6882-13-115 (PMC3698099; doi:10.1186/1472-6882-13-115)
Supplement: Additional file 1: Table S1 — Enzymes that catalyze the modifications in human cells. Figure S1: The distribution of TCM medicinals’ yin-yang scores from their TCM natures. Figure S2: The distribution of TCM medicinals’ yin-yang scores from their TCM flavors. Figure S3: The distribution of TCM medicinals’ yin-yang scores from their TCM natures and flavors. Figure S4: Phylogenetic tree of the 1,208 TCM medicinals. The same plot as Figure 1 in the text except that the labels show the medicinals’ scientific names. Figure S5: Phylogenetic tree of the 1,208 TCM medicinals. The same plot as Figure 1 in the text except that the colors code for yin-yang of the TCM medicinals. The darker the shade, the more yang the medicinal. Figure S6: Phylogenetic tree of the 1,208 TCM medicinals. The same plot as Additional file 1: Figure S2 except that the labels show the scientific names of the TCM medicinals. Figure S7: Proportion of the histone-modifying medicinals among the 230 medicinals that make up the 200 TCM formulas. Figure S8: Hierarchial clustering of the 116 histone-modifying TCM medicinals in the 200 TCM formulas and the 18 histone modifications. Refer to Table S1 for the abbreviations of the modifications. [file 1472-6882-13-115-S1.doc]

**Supplementary Tables**

**Table S1 Enzymes that catalyze the modifications in human cells**

| Position/abbreviation^*^ | Enzymes | Ensembl Protein ID |
| --- | --- | --- |
| DNMT | DNMT  DNMT2  DNMT3A  DNMT3B  DNMT3L | ENSP00000352516  ENSP00000346652  ENSP00000264709  ENSP00000364772  ENSP00000270172 |
| HAT | Histone acetyltransferase 1  Histone acetyltransferase GCN5  Histone acetyltransferase HTATIP  Histone acetyltransferase MORF  Histone acetyltransferase MYST2  Histone acetyltransferase MYST3  Histone acetyltransferase PCAF  Histone acetyltransferase p300  Histone acetyltransferase type B subunit 2  MYST histone acetyltransferase 1, isoform CRA_b | ENSP00000264108  ENSP00000225916  ENSP00000340330  ENSP00000287239  ENSP00000259021  ENSP00000265713  ENSP00000263754  ENSP00000263253  ENSP00000369424  ENSP00000320134 |
| HDAC | Histone deacetylase  Histone deacetylase 1  Histone deacetylase 10  Histone deacetylase 11  Histone deacetylase 2  Histone deacetylase 3  Histone deacetylase 4  Histone deacetylase 5  Histone deacetylase 6  Histone deacetylase 8  Histone deacetylase 9 | ENSP00000309766  ENSP00000362649  ENSP00000216271  ENSP00000295757  ENSP00000357621  ENSP00000302967  ENSP00000264606  ENSP00000225983  ENSP00000334061  ENSP00000362674  ENSP00000262069 |
| H3K4 | Histone H3-K4 methyltransferase  Histone-lysine N-methyltransferase HRX  Histone-lysine N-methyltransferase MLL2  Histone-lysine N-methyltransferase MLL3  Histone-lysine N-methyltransferase MLL4  Histone-lysine N-methyltransferase NSD3  Histone-lysine N-methyltransferase PRDM9  Histone-lysine N-methyltransferase SETD1A  Histone-lysine N-methyltransferase SETD1B  Histone-lysine N-methyltransferase SETMAR | ENSP00000274031  ENSP00000374158  ENSP00000301067  ENSP00000262189  ENSP00000222270  ENSP00000313983  ENSP00000296682  ENSP00000262519  ENSP00000267197  ENSP00000373354 |
| H3K36 | Histone-lysine N-methyltransferase SETD2  Histone-lysine N-methyltransferase SETMAR  Histone-lysine N-methyltransferase, H3 lysine-36 and H4 lysine-20 specific | ENSP00000332415  ENSP00000373354  ENSP00000348031 |
| H3K79 | Histone H3-K79 methyltransferase | ENSP00000221482 |
| H3R17 | Histone-arginine methyltransferase CARM1 | ENSP00000325690 |
| H3K9 | Euchromatic histone-lysine N-methyltransferase 1  Euchromatic histone-lysine N-methyltransferase 2  Histone H3-K9 methyltransferase 1  Histone H3-K9 methyltransferase 2  Histone H3-K9 methyltransferase 4  Histone-lysine N-methyltransferase SETDB2 | ENSP00000298728  ENSP00000259865  ENSP00000365877  ENSP00000346997  ENSP00000357965  ENSP00000326477 |
| H3K27 | Histone-lysine N-methyltransferase EZH1  Histone-lysine N-methyltransferase EZH2  Histone-lysine N-methyltransferase NSD3 | ENSP00000264646  ENSP00000320147  ENSP00000313983 |
| K4K20 | Histone-lysine N-methyltransferase SETD8  Histone-lysine N-methyltransferase SUV420H1  Histone-lysine N-methyltransferase SUV420H2  Histone-lysine N-methyltransferase, H3 lysine-36 and H4 lysine-20 specific | ENSP00000332995  ENSP00000305899  ENSP00000255613  ENSP00000348031 |
| H3K4i | Histone demethylase JARID1A  Histone demethylase JARID1B  Histone demethylase JARID1C  Histone demethylase JARID1D  Lysine-specific histone demethylase 1 | ENSP00000372265  ENSP00000235790  ENSP00000364550  ENSP00000322408  ENSP00000246149 |
| H3K36i | [Histone-H3]-lysine-36 demethylase 1A  [Histone-H3]-lysine-36 demethylase 1B | ENSP00000309302  ENSP00000366271 |
| H3R2 and H4R3 | Histone arginine demethylase JMJD6 | ENSP00000302916 |
| H3K27i | Histone demethylase JMJD3  Histone demethylase UTX  Histone demethylase UTY | ENSP00000254846  ENSP00000367203  ENSP00000351914 |
| H3S10 | Nuclear mitogen- and stress-activated protein kinase 1  Nuclear mitogen- and stress-activated protein kinase 2  Inhibitor of nuclear factor kappa-B kinase subunit alpha | ENSP00000261991  ENSP00000294261  ENSP00000359424 |
| H3S10i | MAP kinase phosphatase 1 | ENSP00000239223 |
| ATP | BRM  BAF60A  BAF60B  BAF60C  BRG1  ACF1  CHRAC-15  CHRAC17  p325 subunit of RSF chromatin-remodeling complex  SNF2H  NURF55  ATP-dependent helicase CHD1  ATP-dependent helicase CHD2  ATP-dependent helicase CHD9  INO80_HUMAN  SRCAP_HUMAN | ENSP00000265773  ENSP00000228253  ENSP00000318451  ENSP00000262188  ENSP00000350720  ENSP00000371859  ENSP00000220913  ENSP00000363286  ENSP00000311513  ENSP00000283131  ENSP00000362592  ENSP00000284049  ENSP00000311700  ENSP00000219084  ENSP00000355356  ENSP00000343042 |

^*^ K, lysine; R, arginine; S, serine; H3K4, H3K4 methylation; H3K4i, H3K4 demethylation; H3S10, H3S10 phosphorylation; H3S10i, H3S10 dephosphorylation

**Supplementary Figures**

**Figure S1**


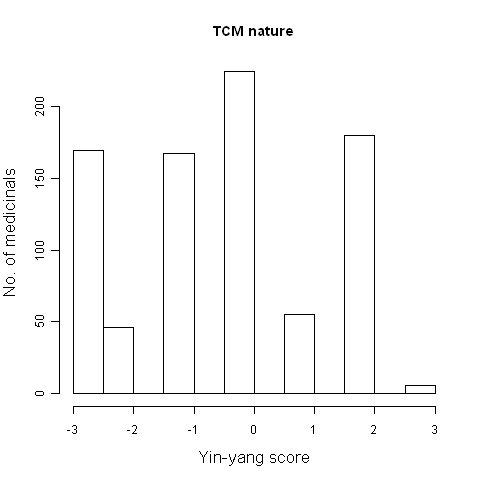


**Figure S1:** The distribution of TCM medicinals’ yin-yang scores from their TCM natures

**Figure S2**


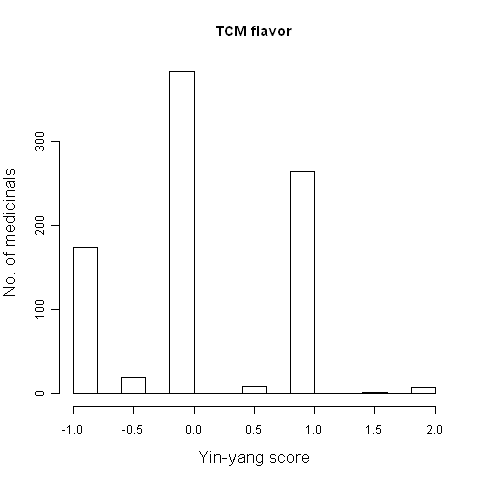


**Figure S2:** The distribution of TCM medicinals’ yin-yang scores from their TCM flavors

**Figure S3**


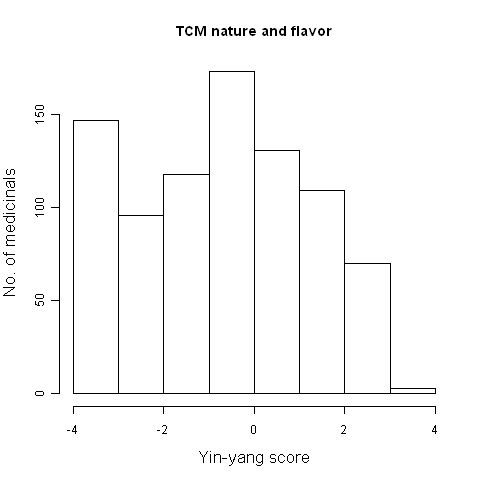


**Figure S3:** The distribution of TCM medicinals’ yin-yang scores from their TCM natures and flavors

**Figure S4**

**
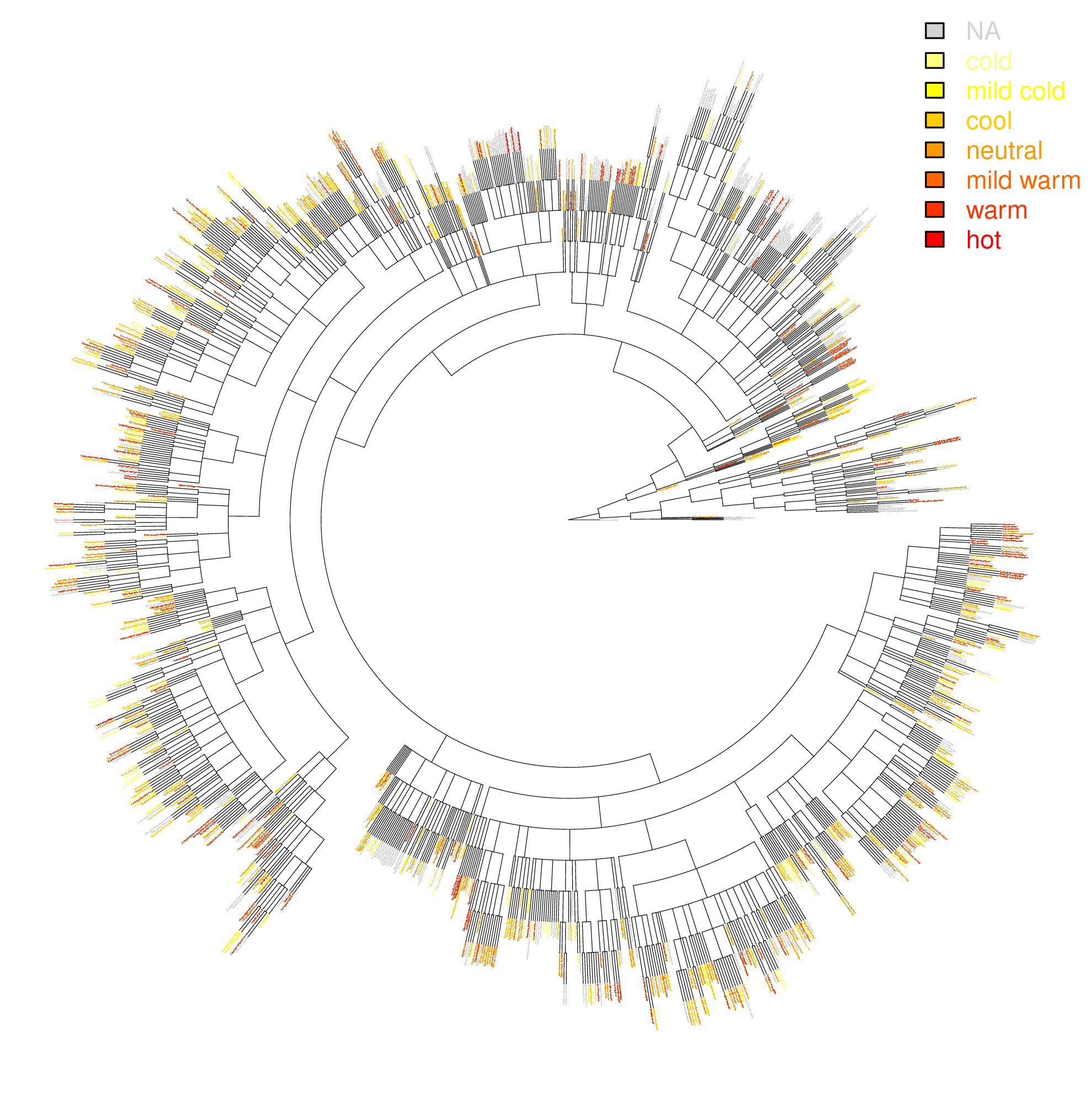
**

**Figure S4: Phylogenetic tree of the 1,208 TCM medicinals.** The same plot as Fig. 1 in the text except that the labels show the medicinals’ scientific names.

**Figure S5**

**
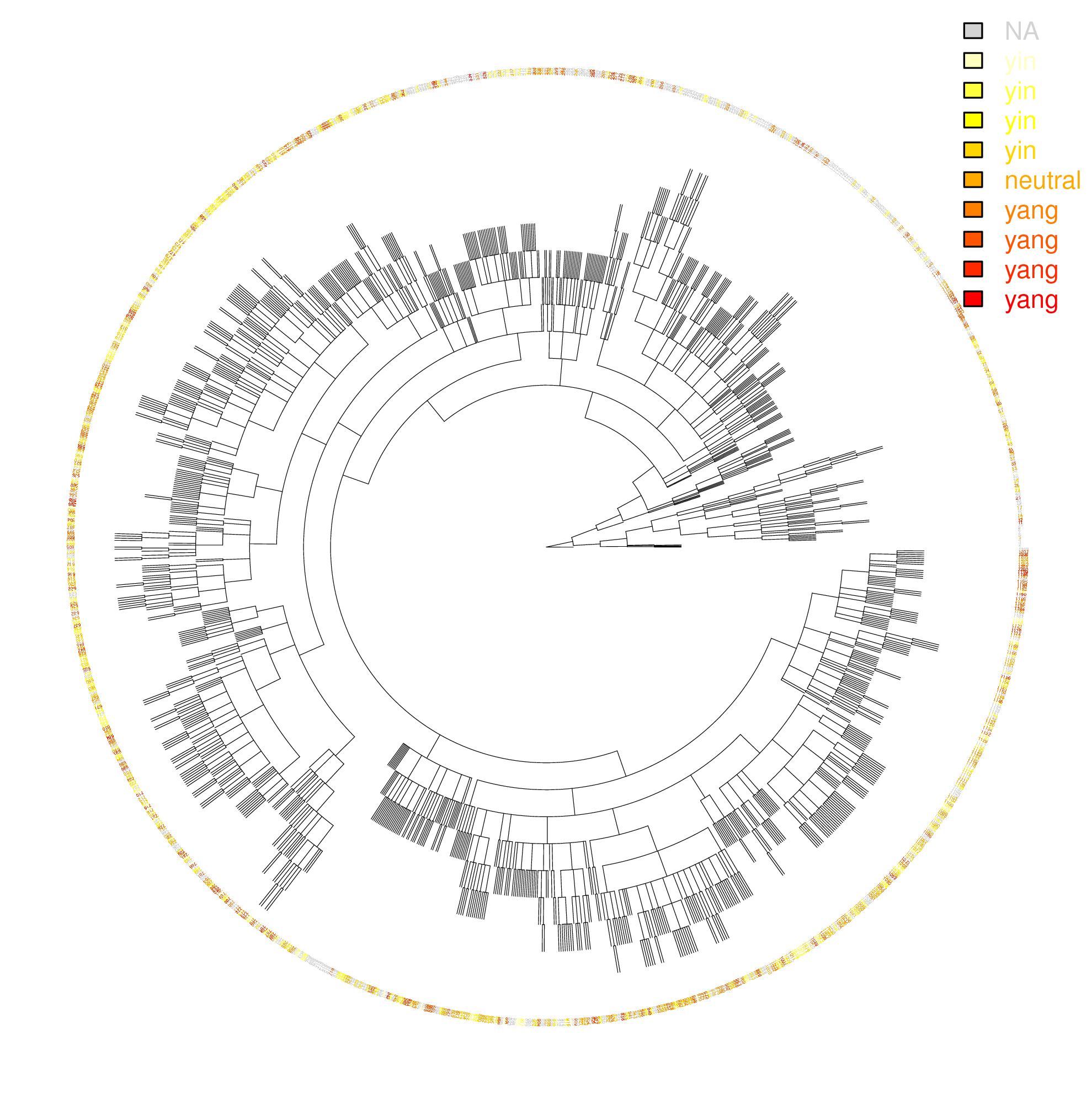
**

**Figure S5: Phylogenetic tree of the 1,208 TCM medicinals.** The same plot as Fig. 1 in the text except that the colors code for yin-yang of the TCM medicinals. The darker the shade, the more yang the medicinal.

**Figure S6**

**
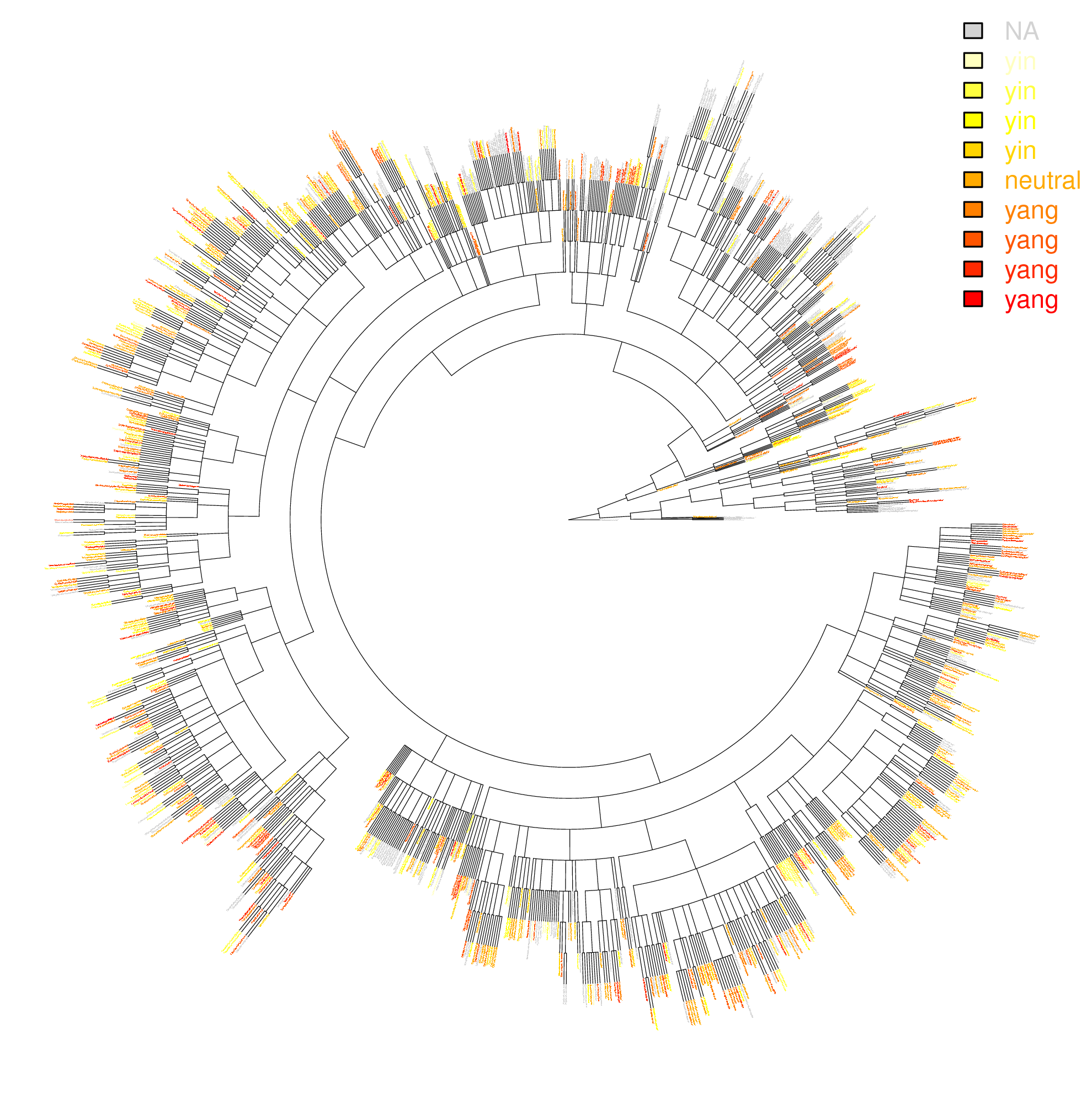
**

**Figure S6: Phylogenetic tree of the 1,208 TCM medicinals.** The same plot as Fig. S2 except that the labels show the scientific names of the TCM medicinals.

**Figure S7**

**
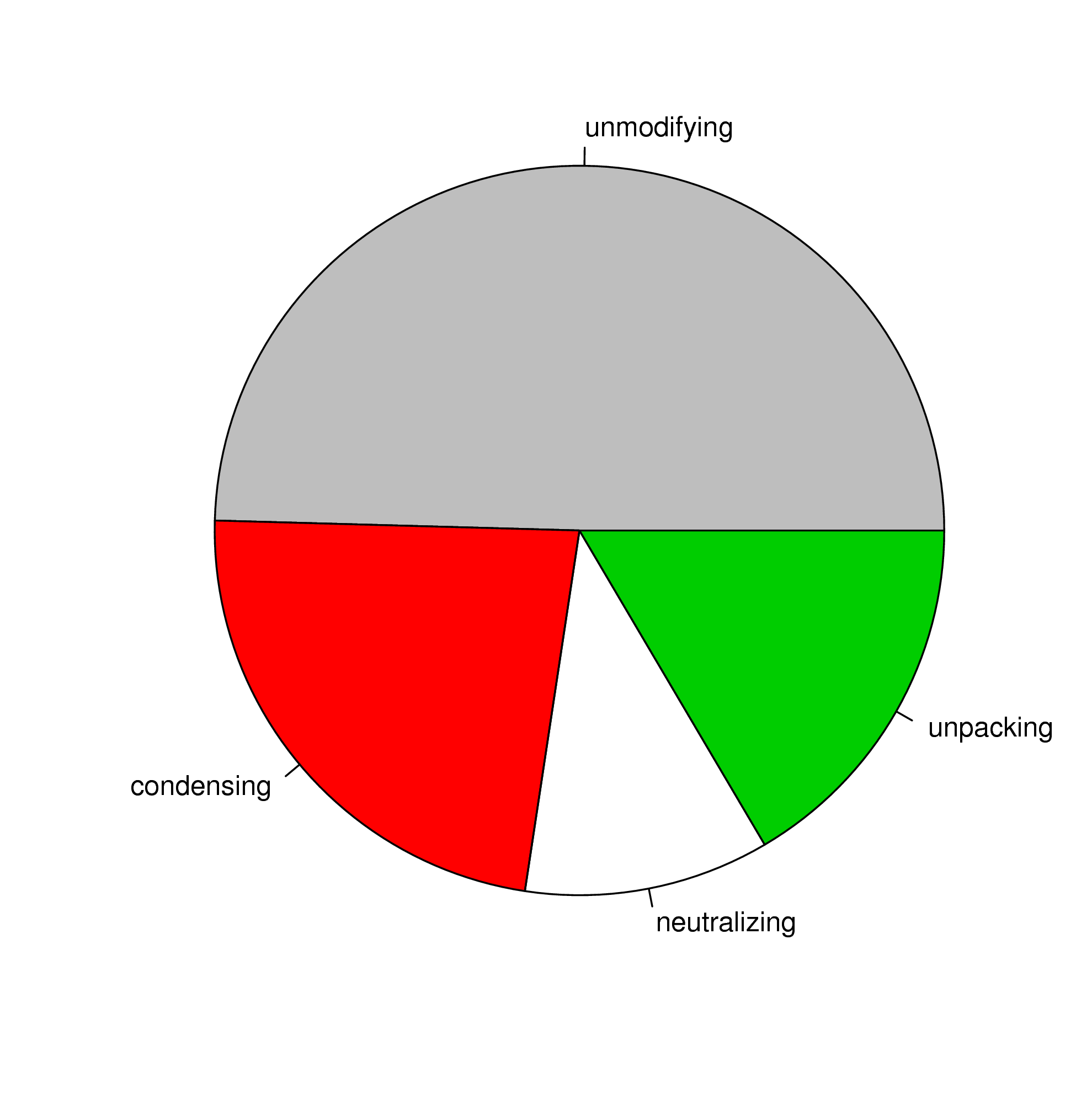
**

**Figure S7: Proportion of the histone-modifying medicinals among the 230 medicinals that make up the 200 TCM formulas.**

**Figure S8**

**
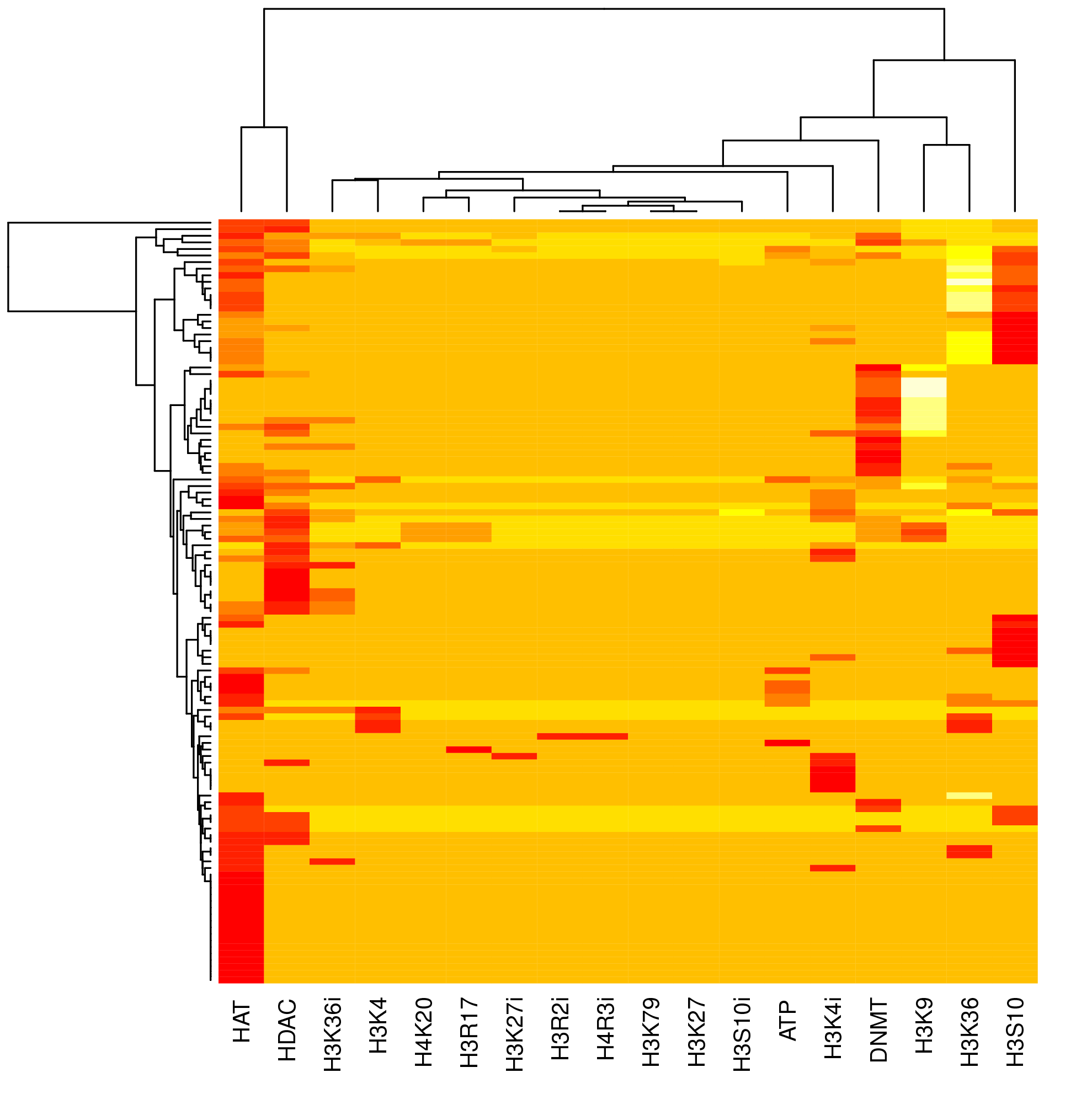
**

**Figure S8: Hierarchial clustering of the 116 histone-modifying TCM medicinals in the 200 TCM formulas and the 18 histone modifications.** Refer to Table S1 for the abbreviations of the modifications.
